# Enhanced NIR Electrochromic Properties of Corannulene-(triphenylamine)_5_ and EDOT-Derived Polymers via Electrochemical Layer-by-Layer Polymerization Compared to Copolymerization

**DOI:** 10.1021/acsami.4c21400

**Published:** 2025-02-05

**Authors:** Chiao-Ling Yu, Shyh-Chyang Luo

**Affiliations:** Department of Materials Science and Engineering, National Taiwan University, No. 1, Sec. 4, Roosevelt Road, Taipei 10617, Taiwan

**Keywords:** electrochromism, corannulene, EDOT, electropolymerization, layer-by-layer polymerization

## Abstract

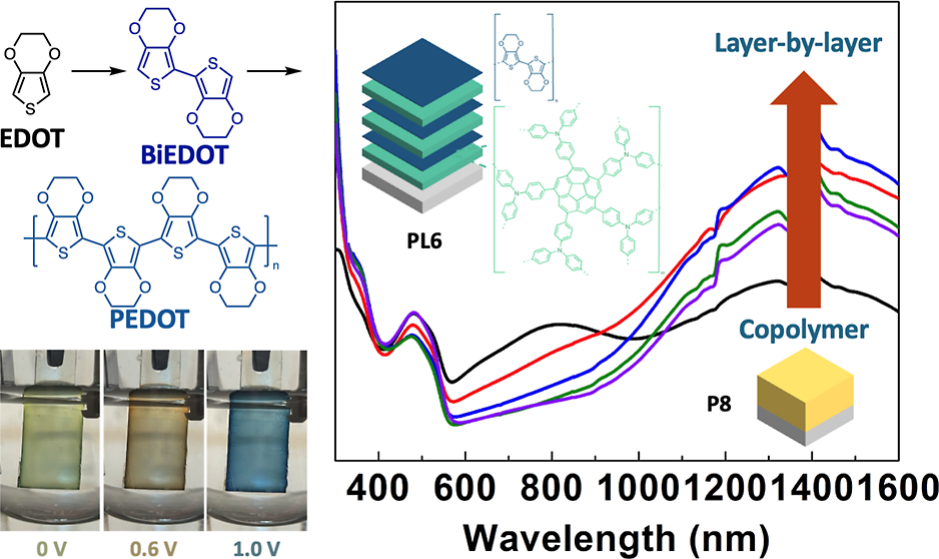

This study explores the electrochemical and electrochromic properties of polymers derived from corannulene-(triphenylamine)_5_ (CT) and 3,4-ethylenedioxythiophene (EDOT) through electrochemical copolymerization and layer-by-layer polymerization techniques. To address the high oxidation potential of EDOT, an EDOT dimer (BiEDOT) was synthesized, which facilitated successful copolymerization with CT. The resulting copolymer films, P10 and P8, exhibit enhanced near-infrared (NIR) electrochromic properties, with P8 showing better performance in NIR absorption. Comparative analysis with layer-by-layer films (PLs) reveals distinct electrochemical behaviors with PLs demonstrating higher NIR and lower visible absorbance with improved optical properties. Among PLs, PL6 exhibits the best NIR absorbance at 0.6 V, while the absorbance of the others does not show a significant increase until 0.7 V. Additionally, it also demonstrates excellent switching properties, with only a 3% loss of optical contrast at 1400 nm after 500 switching cycles. The study concluded that layer-by-layer polymerization offers an effective method for constructing PCT–PEDOT electrochromic films, providing better performance and higher NIR absorption than copolymerized films.

## Introduction

The optical characteristics of electrochromic materials can be altered when an electrical potential is applied. This feature can be utilized in various devices such as displays, biosensors, and camouflage systems.^1−3^ Besides being known for their visible wavelength optical properties, their near-infrared (NIR) absorption is particularly important for energy conservation applications, notably in smart windows.^4−6^ By blocking NIR sunlight radiation, these windows help maintain lower indoor temperatures, thus less power is required.^7,8^ In recent years, inorganic materials like tungsten oxide and nickel oxide have been predominantly used for smart windows due to their excellent electrochemical stability and reversibility.^9−12^ Besides inorganic options, conjugated polymers are also popular as organic electrochromic materials.^13−16^ In spite of the poor stability,^1,17^ conjugated polymer-based electrochromic devices have several advantages, including low cost, multiple color changes, fast response times, and adjustable optical properties through modifying the substituents of the polymer chain.^18−24^

Triphenylamine (TPA) derivatives have been widely used as electrochromic materials due to their reversible redox properties, low redox potential, and significant color change between their neutral and oxidized states.^25−27^ The TPA group can be easily electrochemically oxidized to form a radical cation and subsequently dimerized to create tetraphenyl benzidine (TPB), facilitating subsequent polymerization.^28,29^ Compared with chemical polymerization methods, electropolymerization allows for straightforward thin-film coating on ITO glass. Additionally, the TPB unit can act as a redox center for the intervalence charge-transfer mechanism (IVCT), which absorbs in the NIR region.^30,31^ Various systems, including triphenylamine-based polyimides, polyamides, and other conjugated polymers, have been developed.^32−34^

Among the large amounts of systems, corannulene-(triphenylamine)_5_ (CT) was designed and synthesized as an electrochromic material with excellent electrochromic properties and stability.^35^ Corannulene is a fragment of C_60_, which retains a curved molecular structure.^36−38^ Also, it has a much higher solubility in common organic solutions compared to C_60_, allowing for various synthesis routes and further purification.^39−41^ The nonplanar structure enables PCT to form a porous film through electropolymerization. This porous structure can improve the electrochemical properties of the film by providing a larger surface area with more accessible reactive sites compared to a nonporous structure. It also facilitates ion penetration into the film, further enhancing its electrochromic properties.^42−44^ In this study, 3,4-ethylenedioxythiophene (EDOT) was incorporated with CT as a copolymer to enhance the NIR optical properties further. EDOT was selected since poly(3,4-ethylenedioxythiophene), PEDOT, has a low band gap, high electrochemical stability, and outstanding electrochromic performance.^45−50^ Additionally, the resulting PEDOT polymer exhibits a dark blue color in its neutral state. It becomes light blue in its oxidized state,^51^ making it a promising material for smart windows. As a result, the combination of CT and EDOT was designed to yield a conductive polymer film with NIR electrochromism.

**Scheme 1:**
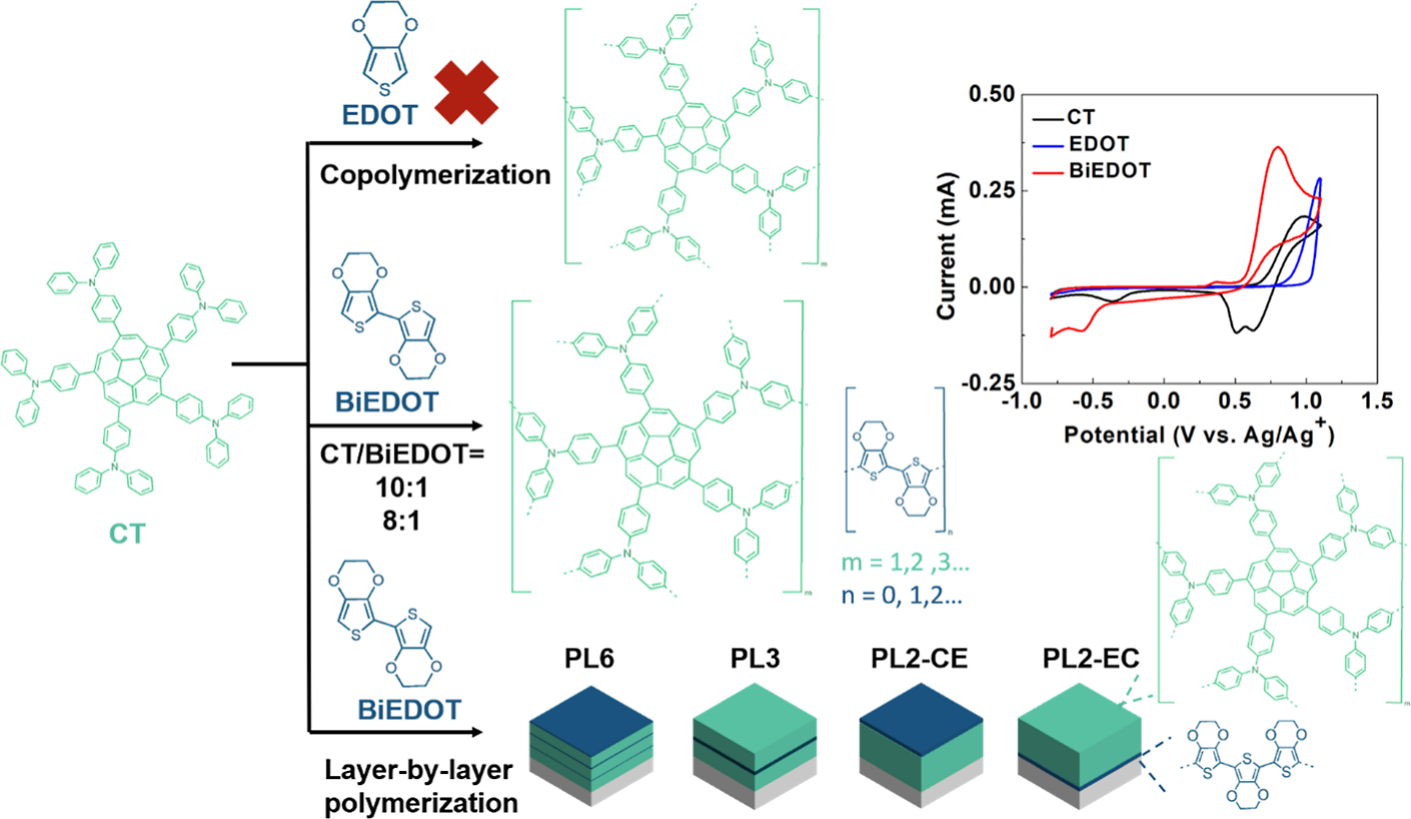
Copolymerization and Layer-by-Layer Polymerization of CT and EDOT/BiEDOT and the Cyclic Voltammograms of CT, EDOT, and BiEDOT Monomers, Showing Their Oxidation Potentials

To combine CT and EDOT, two polymerization strategies can be employed. The first method is electrochemical copolymerization, which involves dissolving CT and EDOT simultaneously in the electrolyte and electropolymerizing them together. The second method is electropolymerizing CT and EDOT layer by layer. The first method offers the advantage of requiring only a single step, providing a more straightforward adjustment to the experimental conditions. However, in this method, it is necessary that the oxidation potentials of the two substances are compatible, which allows them to be oxidized at similar rates. After the electrochemical properties of the monomers were examined, EDOT was found to have an oxidation potential higher than that of CT, making copolymerization unworkable. Therefore, we synthesized its dimer, BiEDOT, which has an oxidation potential similar to that of CT, to facilitate copolymerization. The concentration ratio can be adjusted to achieve efficient copolymerization, as illustrated in Scheme 1. In contrast, the second method involves multiple steps to form layers of different polymers. This method requires careful consideration of the concentration of each monomer in the electrolytes, the number of CV cycles for each monomer, the quantities, and the sequence of the layers. Despite the increased complexity, this method allows a larger disparity in the oxidation potentials of each monomer. Additionally, the variety of possible layer structures offers significant potential for developing new electrochromic properties in the resulting polymer. In this work, CT and BiEDOT were copolymerized in various ratios to identify the optimal ratio for the best electrochromic properties. In addition, four layer-by-layer structures with different sequences of the component layers were constructed. A comparative study of the resulting electrochemical and optical properties of the polymers was conducted to provide a deeper understanding of the mechanism of these two methods.

## Experimental Section

### Monomer Synthesis

All reactions were performed using standard vacuum-line and Schlenk techniques. Workup and purification procedures were carried out with reagent-grade solvents under air. Nuclear magnetic resonance (NMR) spectra were recorded on a Bruker AVIII 500 MHz FT-NMR (1H 500 MHz) spectrometer. Chemical shifts for ^1^H NMR are expressed in parts per million (ppm) relative to CD_2_Cl_2_ (δ 5.32 ppm) and (CD_3_)_2_S = O (δ 2.54 ppm). Data are reported as follows: chemical shift, multiplicity (s = singlet, d = doublet, t = triplet, dt = doublet triplet, and m = multiplet), coupling constants (Hz), and integration. According to the reported procedures, CT^35^ was synthesized as a green solid. ^1^H NMR (500 MHz, CD_2_Cl_2_, δ): 7.96–7.93 (m, 1H), 7.63 (d, *J* = 8.6 Hz, 2H), 7.33–7.27 (m, 4H), 7.18 (dd, *J* = 8.0, 3.6 Hz, 6H), 7.07 (t, *J* = 7.3 Hz, 2H), which is shown in Figure S1. 2,2′-Bis(3,4-ethylenedioxy)thiophene (BiEDOT) was synthesized following a previous study.^52^^1^H NMR (500 MHz, DMSO, δ): 6.57 (s, 1H), 4.36–4.33 (m, 2H), 4.27–4.25 (m, 2H), which is shown in Figure S2.

### Electrochemical Polymerization and Experiments

Propylene carbonate (PC) was distilled with CaO before used. All of the other chemicals were used without further purification. Electrochemical experiments were performed using an Autolab PGSTAT128N potentiostat (Metrohm, The Netherlands) in a three-electrode setting, equipped with platinum as the counter electrode and Ag/Ag^+^ (10 mM of AgNO_3_ and 100 mM TBAP in acetonitrile) as the reference electrode. The electropolymerization experiments were carried out with a mixed solvent (dichloromethane/chlorobenzene) containing 0.75 mM CT and 100 mM TBAPF_6_ and cycling potential scans were performed from −0.8 V to +1.1 V versus Ag/Ag^+^ at a scan rate of 100 mV s^–1^. Cyclic voltammetry (CV) of the conjugated polymer films was conducted in dichloromethane (DCM) solution containing 100 mM TBAPF_6_ at different scan rates within the potential range from −0.8 V to +1.1 V versus Ag/Ag^+^.

### Polymer Film Characterization

Spectroelectrochemistry of the conjugated polymer films was conducted in PC with 100 mM TBAPF_6_ under applied potential scans from 0.0 to 1.1 V versus Ag/Ag^+^. X-ray photoelectron spectroscopy (XPS) data were recorded on a ULVAC-PHI (Quantes) XPS instrument with a dual scanning X-ray source (a hard X-ray source [Cr Kα] and a soft X-ray source [Al Kα]), and 1 × 1 cm^2^ ITO glasses or gold disks were used as substrates. The morphologies of the polymer films were observed through FE-SEM (JeoL JSM-7800F Prime) at an accelerating voltage of 5.0 kV. The static contact angle of water on the polymer films was measured by a contact angle goniometer (Model 100 SB, Sindatek). Each polymer film was measured at least five times to receive average values and standard deviations. UV/vis spectra were recorded on a JASCO V-770 UV–Visible/NIR spectrometer.

## Results and Discussion

### Electropolymerization of CT and EDOT

First, the electrochemical properties of corannulene, CT, and EDOT were assessed prior to electropolymerization. Both compounds were, respectively, dissolved in a DCM/chloroform (1:1) mix solution containing 0.1 M tetrabutylammonium hexafluorophosphate (TBAPF_6_). CV scans were performed from −0.8 to 1.2 V versus Ag/Ag^+^, and the results are presented in Scheme 1 and Figure S3. Corannulene exhibits no redox reactions within this potential range. The oxidation potential of EDOT was found to be 0.9 V, which is significantly higher than that of CT at 0.4 V. Consequently, less EDOT was expected to be deposited on the substrate during CV. Therefore, to promote the electrochemical copolymerization, the concentration of EDOT was increased to 5, 7.5, and 10 times that of the CT concentration. The molar ratios of the two monomers in the resulting polymer films were observed by XPS. The results of the synthesized films (Figure S4) show that none of the three films exhibited a peak in the S 2p spectra, indicating that none of EDOT can be successfully copolymerized with CT even with increasing amounts of EDOT. It is known that higher conjugation leads to a lower band gap.^53^ Therefore, the EDOT dimer (BiEDOT) was synthesized to address the issue by extending the conjugation, thereby lowering the oxidation potential. BiEDOT was synthesized via Ullmann coupling according to the reported procedure.^52^ As shown in Scheme 1, CV of BiEDOT indicates that it oxidizes at a potential 0.4 V lower than that of EDOT, making it feasible for electropolymerization with CT.

### Electropolymerization of CT and BiEDOT

**Figure 1:**
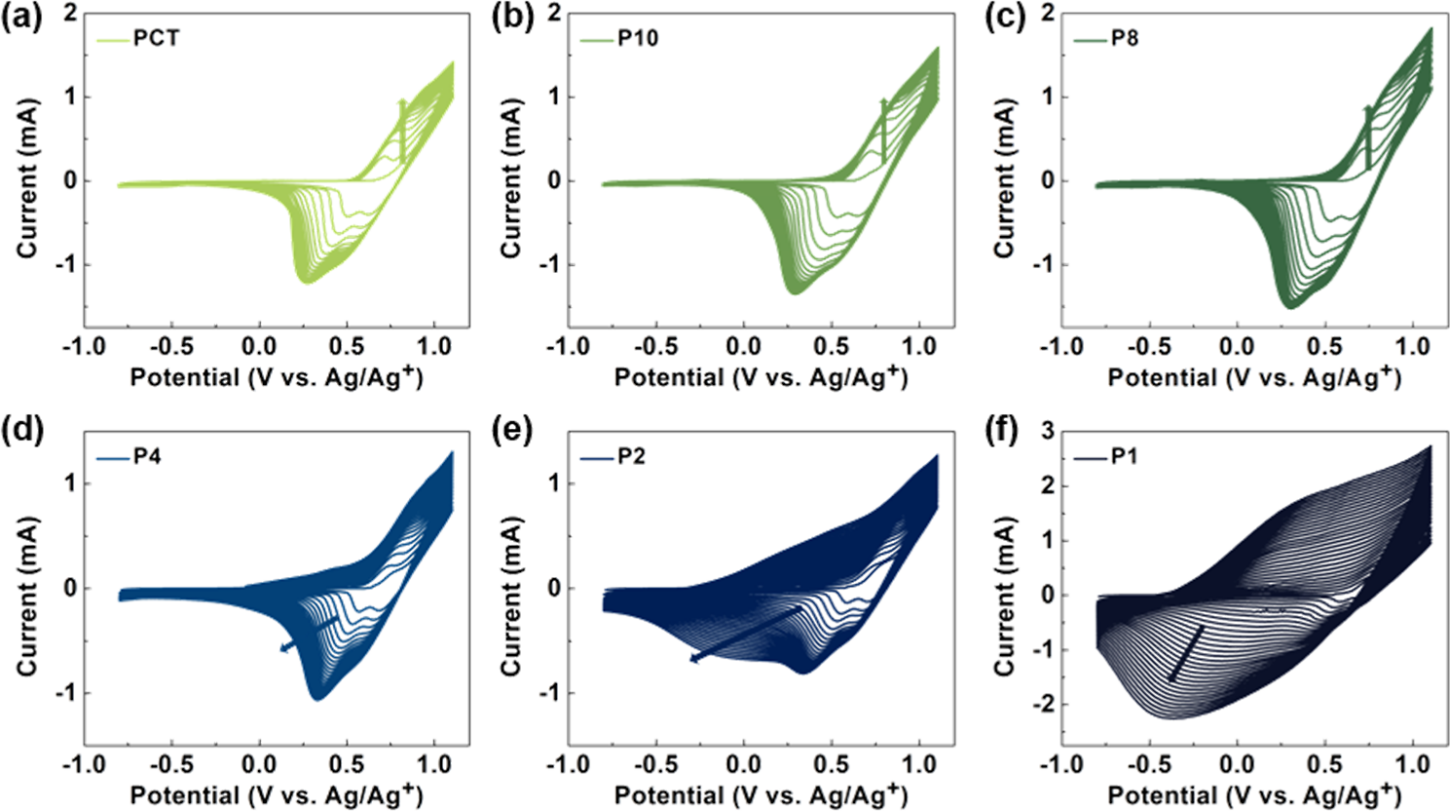
Electropolymerization of (a) PCT, (b) P10, (c) P8, (d) P4, (e) P2, and (f) P1.

0.75 mM CT (0.75 mM) and BiEDOT were dissolved in a DCM/chlorobenzene (1:1) solution containing 0.1 M TBAPF_6_. Six different CT/BiEDOT ratios were tested: 1:0, 10:1, 8:1, 4:1, 2:1, and 1:1, resulting in polymer films named PCT, P10, P8, P4, P2, and P1, respectively. With the CT concentration fixed at 0.75 mM, the amount of BiEDOT increased from PCT to P1. The electropolymerization was conducted using a CV program within the potential range from −0.8 to 1.1 V versus Ag/Ag^+^. As shown in Figure 1a–c, due to the low and similar concentrations of BiEDOT in the electrolytes, PCT, P10, and P8 exhibit similar CV results. During the first oxidative scan, an oxidation peak indicating the formation of a TPA radical cation can be observed, followed by two reduction peaks during the reductive scan. In the second scan, a shoulder around 0.5 V appears, indicating the formation of tetraphenylbenzidine (TPB). This is resulted from the reactive TPA radical cation bonding with other monomer molecules, along with the coupling of BiEDOT, leading to polymer chain formation.^54,55^ In subsequent scans, the increasing redox current indicates the growing amount of polymer deposited on the substrate, while the lower oxidation potential reflects extended conjugation, both confirming successful electropolymerization. For P4, P2, and P1, as shown in Figure 1d–f, the gradual increase in BiEDOT concentration leads to distinct electropolymerization behavior compared to that of PCT, P10, and P8. The reductive current in the potential range of 0.2 V to −0.8 V increases, which is attributed to the redox process of BiEDOT. Additionally, the oxidation potential during electropolymerization significantly decreases, indicating an increase in conjugation length. Both phenomena become more pronounced with higher BiEDOT concentrations.

### Electrochemical Properties of the CT-BiEDOT Copolymer

**Figure 2:**
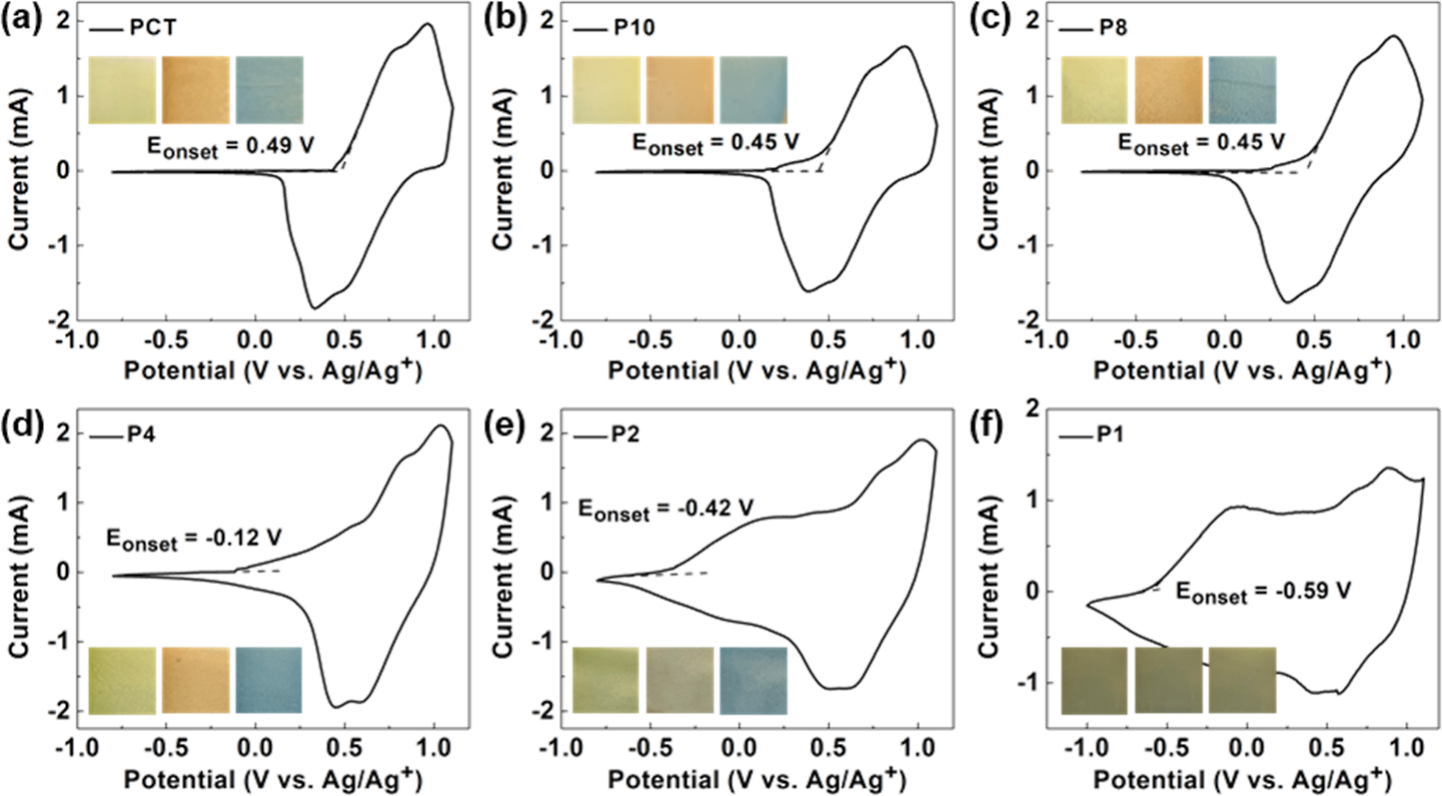
CV diagrams in DCM at the neutral state for (a) PCT, (b) P10, (c) P8, (d) P4, (e) P2, and (f) P1 films. The inset displays the colors of each film at applied potentials of 0, 0.6, and 1.0 V (from left to right).

The electrochemical properties of polymer films were tested using CV in DCM containing 0.1 M TBAPF_6_ under a potential range from −0.8 to 1.1 V versus Ag/Ag^+^ as shown in Figure 2. The onset oxidation potentials of PCT, P10, P8, P4, P2, and P1 were calculated from the CV diagram as 0.49, 0.45, 0.45, −0.12, −0.42, and −0.59 V, respectively. Since the concentration of BiEDOT in P10 does not differ significantly from that in P8, they both have similar oxidation potentials, which are lower than that of PCT. However, when the concentration of BiEDOT increases significantly, both onset potentials and the shapes of the CV voltammograms of P4, P2, and P1 are changed, displaying more PEDOT-like characteristics with a broadband. As a result, the structure of the polymer films can be distinguished from PCT–PEDOT homopolymer blends, in which two distinct oxidation peaks corresponding to PCT and PEDOT should be observed at their respective oxidation potential. The change in the onset potential for each film also provides evidence supporting the (CT)_*m*_–(BiEDOT)_*n*_ structure, as only the incorporation of an additional -(BiEDOT)_*n*_- segment bonded to a -(CT)_*m*_- segment would affect the total conjugation of the film. The decreasing onset oxidation potential with increasing BiEDOT concentration indicates a higher HOMO level from P1 to PCT. The HOMO levels of each polymer film were calculated and are presented in Table 1. The inset of Figure 2 displays the color of each polymer film at 0, 0.6, and 1.0 V; since PEDOT exhibits a dark blue color at 0 V, the amount of PEDOT in the polymer film was controlled to maintain sufficient transmittance of visible light. As shown in Figure 2, P4, P2, and P1 appear too dark at 0 V, resulting in small transmittance and visible change between their neutral and oxidized states compared with PCT, P10, and P8. As a result, P10 and P8 were selected for further characterization.

**Table 1:** Information of PCT, P10, and P8 Film

sample	*E*_onset_ (V)	CT/BiEDOT feeding ratio	CT/BiEDOT repeat unit ratio	HOMO level (eV)^a^
PCT	0.49	1:0	1:0	–5.38
P10	0.45	10:1	15:1	–5.34
P8	0.45	8:1	7:1	–5.34
P4	–0.12	4:1		–4.77
P2	–0.42	2:1		–4.47
P1	–0.59	1:1		–4.30

a HOMO levels were calculated from the onset potentials, which were referenced to ferrocene (*E*_1/2 ferrocene_ = 0.27 V)^30^; *E*_HOMO_ = −(*E*_onset_ – *E*_1/2 ferrocene_)–5.16.^57^

**Figure 3:**
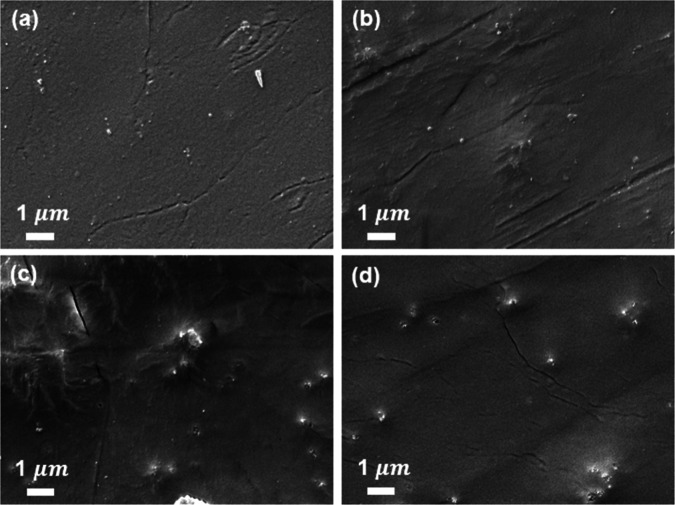
SEM images of (a) PCT, (b) P10, (c) P8, and (d) PEDOT films.

Scanning electron microscopy (SEM) images (Figure 3) and contact angle measurements (Figure S5) of each film confirm the successful formation of thin films on the substrate. Additionally, the ratios of CT to BiEDOT in the polymer films were determined by XPS analysis (Figure S6), yielding ratios of 15:1 for P10 and 7:1 for P8. The difference between the feed ratio and the measured ratio obtained in XPS can be attributed to the slower electropolymerization rate of the less concentrated monomer owing to less accessible radical cations.^56^ Additionally, the slightly lower oxidation potential of BiEDOT compared with CT further influences the electropolymerization process. Together, the concentration and electrochemical effects determine the resulting ratio of CT to BiEDOT in the copolymer. The results highlight a competitive relationship between CT and BiEDOT during the electropolymerization.

The redox behavior of PCT, P10, and P8 was tested by CV in PC containing 0.1 M TBAPF_6_ under a potential range from −0.8 to 1.1 V versus Ag/Ag^+^ (Figure S7). The results are similar among all three polymers, displaying one redox couple. During the oxidative scan, the colors of the films changed from light yellow to red and then to dark blue. The CV procedure was further conducted at different scan rates (20, 50, 80, 100, 200, and 300 mV/s) to investigate the kinetics of the redox process. Both current of the oxidation peak and the reduction peak increase linearly with an increasing scan rate, indicating a diffusion-controlled redox reaction.

### Spectroelectrochemical Characterization of the CT-BiEDOT Copolymer

**Figure 4:**
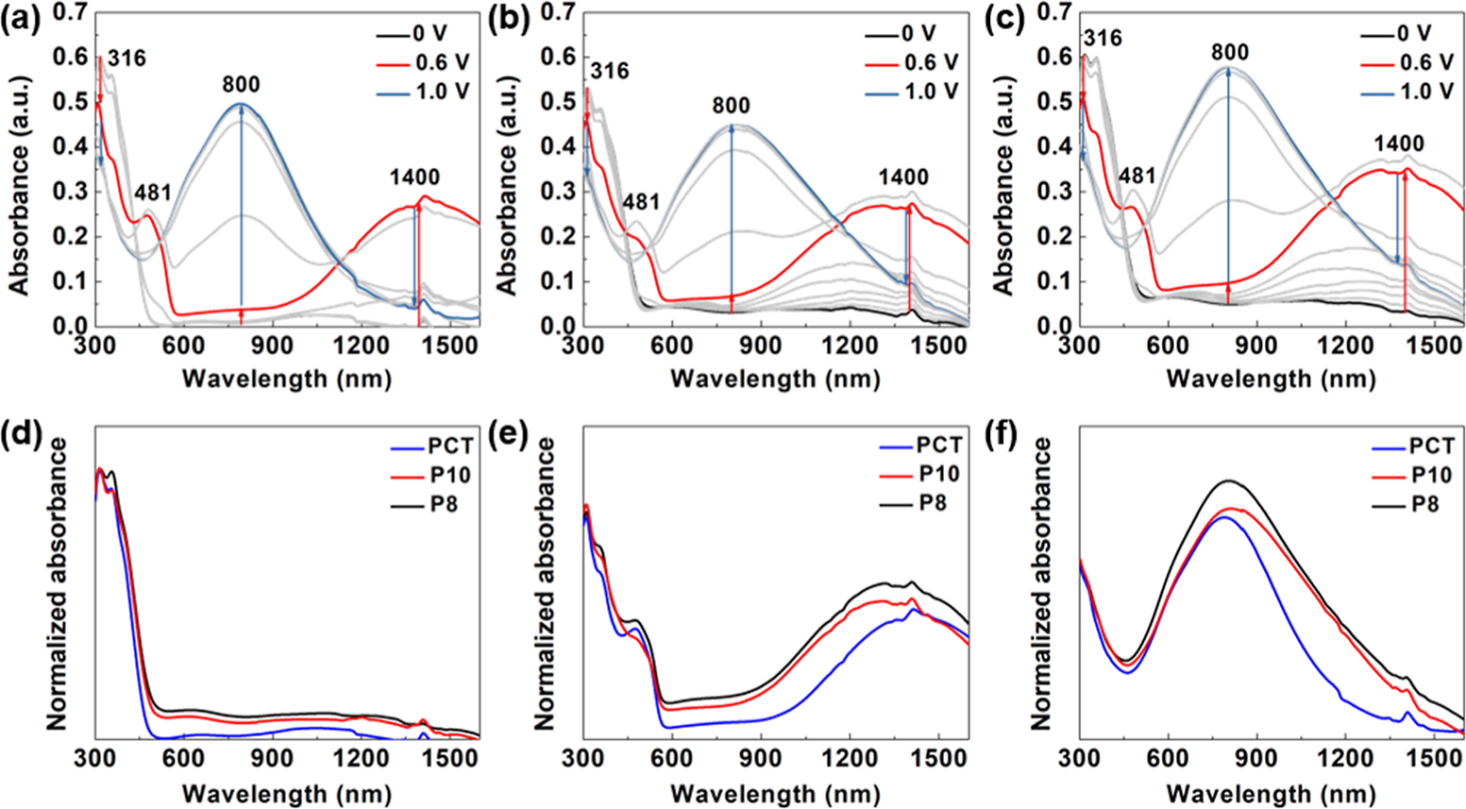
Spectroelectrochemistry of (a) PCT, (b) P10, and (c) P8 with applied potentials from 0 to 1.1 V and the normalized absorbance of PCT, P10, and P8 under (d) 0 V, (e) 0.6 V and (f) 1.0 V.

The NIR electrochromic characteristics of the polymer films were investigated by measuring the absorption across a continuous spectrum under different applied potentials. The measurements were conducted in PC containing 0.1 M TBAPF_6_, under potentials ranging from 0 to 1.1 V versus Ag/Ag^+^ with a 0.1 V interval. As illustrated in Figure 4, in the neutral state (0 V), PCT, P10, and P8 display an absorption peak at 316 nm and a shoulder at 362 nm, originating from the π → π* transition of PCT. While all three films exhibit low absorbance in both visible and NIR region, there is still a small increase in absorbance from PCT to P10, and to P8, attributed to the presence of BiEDOT. With increasing applied potential, the absorption at 316 nm decreases. PCT shows no change in absorption in the NIR region until 0.5 V, whereas absorption of P10 and P8 increases with the applied potential, indicating the effect of BiEDOT. When a potential of 0.6 V is applied, a peak at 481 nm and a sharp enhancement of the absorption band around 1400 nm appear in PCT, P10, and P8, resulted from the formation of the TPB radical cation. Notably, in PCT, the highest absorption at 1400 nm is observed at 0.6 V. However, the absorption of P10 and P8 continues to increase with an applied potential up to 0.7 V while maintaining considerable visible transparency. Meanwhile, the colors of the films change from light yellow to red. Upon further increase of applied potential, the absorption at 400 nm and the band around 1400 nm decrease. An absorption band centered at 800 nm begins to emerge at 0.7 V. This is attributed to the formation of a TPB dication, causing the color of the films to change from red to dark blue.

The optical performances of PCT, P10, and P8 under 0, 0.6, and 1.0 V were compared. Although P10 and P8 can reach higher absorbance at 0.7 V, there is only a slight difference between their performances at 0.6 and 0.7 V. Therefore, to compare with PCT, the absorbance in the NIR region was monitored at 0.6 V. Since the absorbance of a film is proportional to its thickness, the absorbance was normalized with respect to 316 nm, the characteristic peak of CT. By fixing the CT content, the effect of BiEDOT could be emphasized. The normalized results under different applied potentials are shown in Figure 4d–f. Under each applied potential, the wavelength of absorption red shifts and the absorbance increases with the increasing content of BiEDOT. The results are also consistent with the (CT)_*m*_–(BiEDOT)_*n*_ structure.

**Figure 5:**
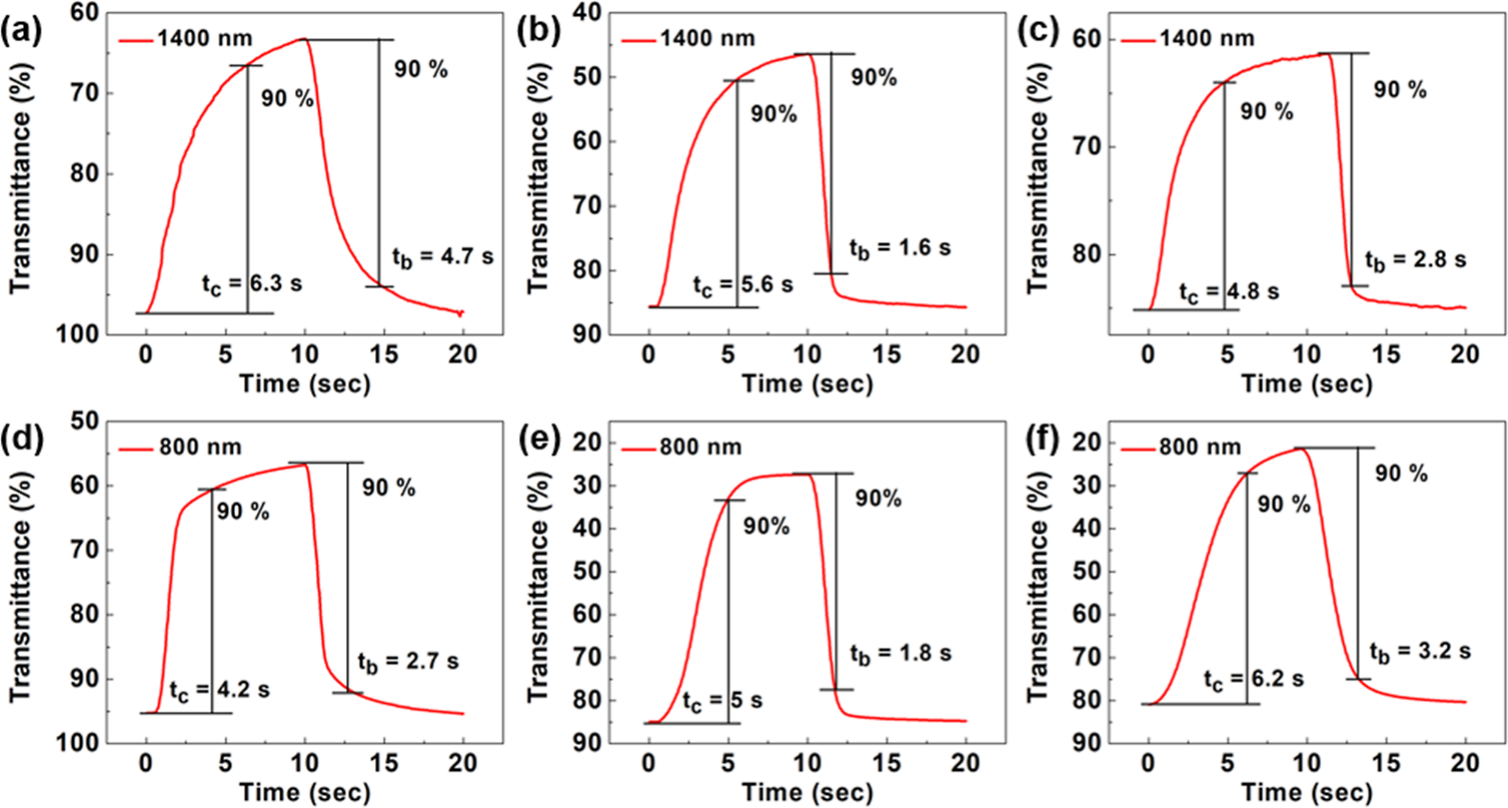
Coloration and bleaching time (*t*_c_ and *t*_b_) of (a) PCT, (b) P10, and (c) P8 at 1400 nm. (d) PCT, (e) P10, and (f) P8 at 800 nm.

The switching time of the films was tested by a square-wave potential sweep from 0 to 0.6 and 1.0 V versus Ag/Ag^+^ with a pulse time of 10 s (Figure 5). The transmittance of the films was monitored at 1400 and 800 nm when applying 0.6 and 1.0 V, respectively. The coloring time (the time taken to reach 90% optical change from the neutral state to the coloring state), *t*_c_, of PCT, P10, and P8 under 0.6 V is 6.3, 5.6, and 4.8 s, respectively. The bleaching times, *t*_b_, are 4.7, 1.6, and 2.8 s, respectively. Under 1.0 V, the *t*_c_ values of PCT, P10, and P8 are 4.2, 5, and 6.2 s, respectively, and *t*_b_ values are 2.7, 1.8, and 3.2 s, respectively. Moreover, the cycling stability of the films was also tested (Figure S8). After 200 switching cycles between 0 and 0.6 V, PCT loses 4% of the optical contrast at 1400 nm, while P10 and P8 maintain high stability, both with 1% of loss. In the case of 800 nm, after 200 switching cycles between 0 and 1.0 V, PCT loses 7% of the optical contrast, whereas P10 and P8 lose 1% and 0.4%, respectively. Moreover, stability at 1400 nm for P8 under 500 switching cycles between 0 and 0.6 V was further conducted. After 500 switching cycles, P8 loses only 3% of the optical contrast. This copolymer shows extraordinary stability.

### Electrochemical Impedance Spectroscopy of the CT-BiEDOT Copolymer

**Figure 6:**
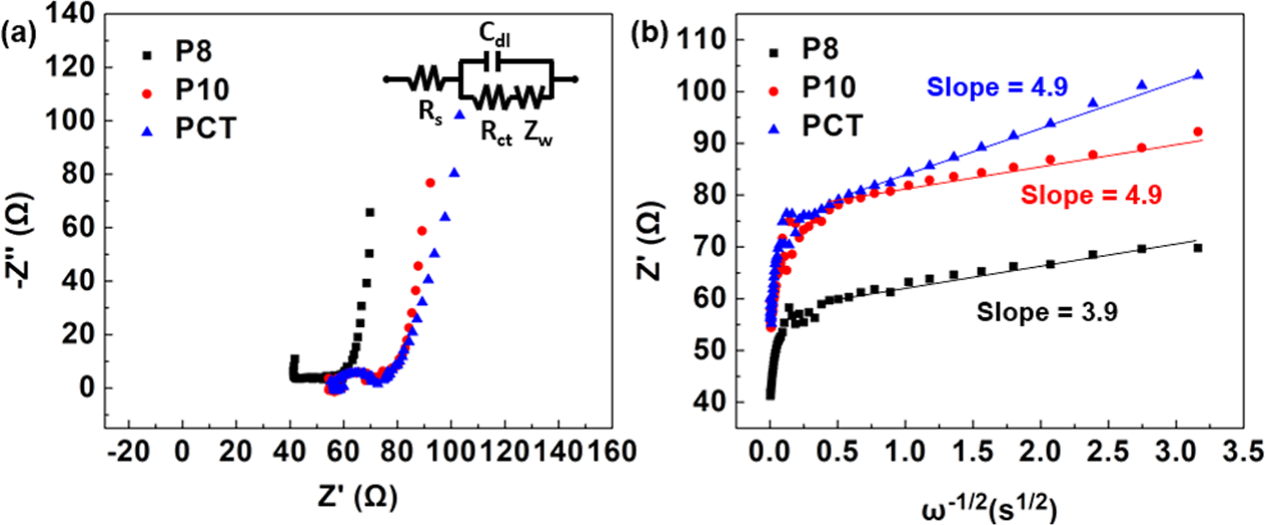
Nyquist plot and equivalent circuit (a) and *Z*′ versus ω^–1/2^ graph (b) of PCT, P10, and P8.

EIS was conducted at 0.6 V over a frequency range of 100 kHz to 0.1 Hz in PC containing 0.1 M TBAPF_6_ to study the charge transfer and diffusion characteristics of the copolymer. The results, along with the corresponding equivalent circuit, are shown in Figure 6a. The semicircle observed in the high-frequency region represents the charge-transfer resistance (*R*_ct_). CT, P10, and P8 exhibit similar R_ct_ values, indicating comparable reaction rates. In the low-frequency region, the line reflects diffusion within the film, with a slope close to 45°, suggesting a Warburg diffusion behavior. The diffusion characteristics are quantified by the Warburg coefficient (σ), expressed as
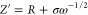
1

Using eq 1, a plot of *Z*′ versus ω^–1/2^ was created, as shown in Figure 6b. The slope of this plot represents the Warburg coefficient for each film.^58^ All three films exhibit similar and small Warburg coefficients with a slight decrease from PCT to P10 and P8. These results align well with the switching behavior observed for each film.

### Layer-by-Layer Polymerization

A layer-by-layer group (PLs) was conducted by coating CT and BiEDOT separately to compare the electrochemical and electrochromic properties with those of the copolymerization method. The polymerization was carried out using CV under the same conditions as those in the previous experiment. To maintain the consistency of the two methods, the feeding molecule ratio of CT and BiEDOT in PLs was controlled to match that in P10 and P8, which are 10:1 and 8:1, respectively. The concentration of BiEDOT was adjusted to be the same as CT and nine scans of CT were performed for every scan of BiEDOT for the convenience of the calculation. By keeping the total number of scans constant under this restriction, four samples were designed: (PCT–PEDOT)_3_, PCT–PEDOT-PCT, PCT–PEDOT, and PEDOT–PCT, denoted as PL6, PL3, PL2-CE, and PL2-EC, respectively. The number in the name refers to the number of alternating layers, and PL2-CE and PL2-EC denote the coating sequence. That is, in PL6, there are six layers of alternating PCT and PEDOT. In PL3, one layer of PCT is followed by a layer of PEDOT and another layer of PCT. In PL2-CE, PCT is deposited first, followed by PEDOT, while in PL2-EC, PEDOT is deposited first and then PCT. These four structures were designed to compare with P8 while also examining the impact of layer number and the sequence of polymer deposition on the resulting electrochemical and electrochromic properties. The preparation of PLs is shown in Figure S9.

### Electrochemical Properties of the Layer-by-Layer Structure

**Figure 7:**
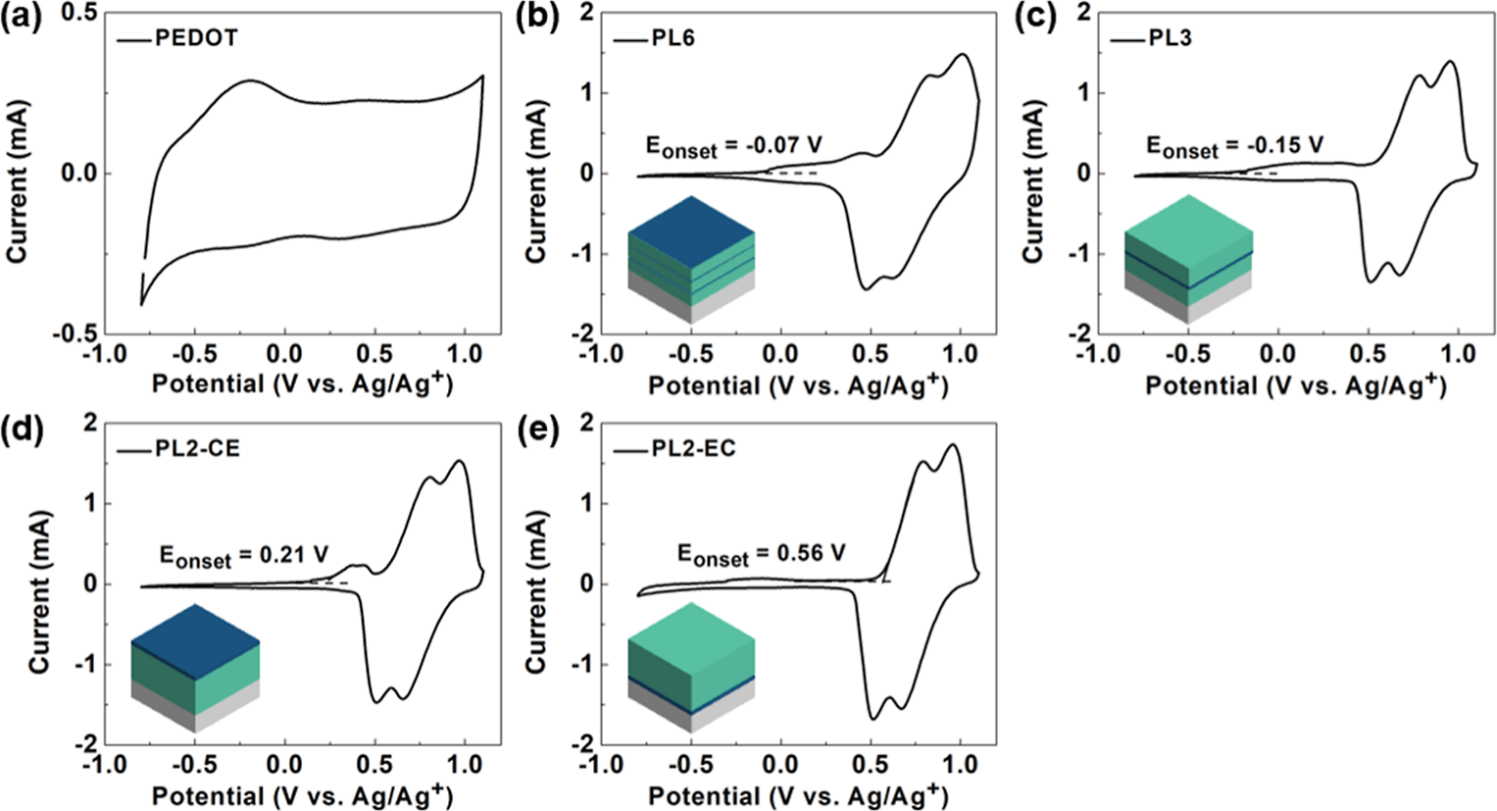
CV diagrams for (a) PEDOT, (b) PL6, (c) PL3, (d) PL2-CE, and (e) PL2-EC films in DCM. The cycles of PCT and PEDOT were fixed at a 9:1 ratio and distributed across 6, 3, 2, and 2 sequences for PL6, PL3, PL2-CE, and PL2-EC, respectively. The difference between PL2-CE and PL2-EC lies in the coating order: for PL2-CE, PCT was coated first, whereas for PL2-EC, PEDOT was coated first.

The redox behavior of PLs was also tested using CV in PC containing 0.1 M TBAPF_6_ under a potential range from −0.8 to 1.1 V versus Ag/Ag^+^ (Figure S10). They also show a diffusion-controlled reaction. In addition, the electrochemical properties of PL6, PL3, PL2-CE, and PL2-EC were tested by CV in DCM containing 0.1 M TBAPF_6_ under a potential range from −0.8 to 1.1 V versus Ag/Ag^+^ as shown in Figure 7. PL2-CE and PL2-EC, which exhibit the most contrasting behavior, are discussed first. PL2-CE displays two distinct oxidation peaks attributed to PEDOT and PCT, whereas PL2-EC shows only one peak, which is contributed solely by PCT. This difference is due to the sequence of polymerization. In PL2-EC, in which PCT was coated on top of PEDOT, during oxidation, although electrons are extracted from PEDOT to the electrode, the PEDOT layer cannot be doped until the outer PCT layer is also oxidized, enabling the ions to penetrate into the full stacking.^59^ Since the oxidation potential of PCT is much higher than that of PEDOT (Figure 7a), the entire film oxidizes simultaneously, resulting in a single oxidation peak with an onset potential of 0.56 V. In this case, PEDOT acts as part of the electrode, with a consistent background current in CV. Conversely, in PL2-CE, the outer PEDOT layer can be oxidized when electrons are extracted from the electrode, and the PCT layer subsequently oxidizes when its onset potential is reached. Thus, PL2-CE shows two distinct peaks during oxidation. For PL3, although the outer layer is PCT, its thickness is half that of PL2-EC. Therefore, PF_6_^–^ ions can penetrate into the PEDOT layer, albeit with some impediment. As a result, the oxidation peak due to PEDOT is less obvious compared to PL2-CE. Lastly, since PL6 has PEDOT as its outer layer, it also shows two oxidation peaks. Due to the consecutive PEDOT–PCT layers, the oxidative behavior of the PEDOT layer manifests as a band before the peak, representing the three separate PEDOT layers.

### Spectroelectrochemical Characterization of the Layer-by-Layer Structure

**Figure 8:**
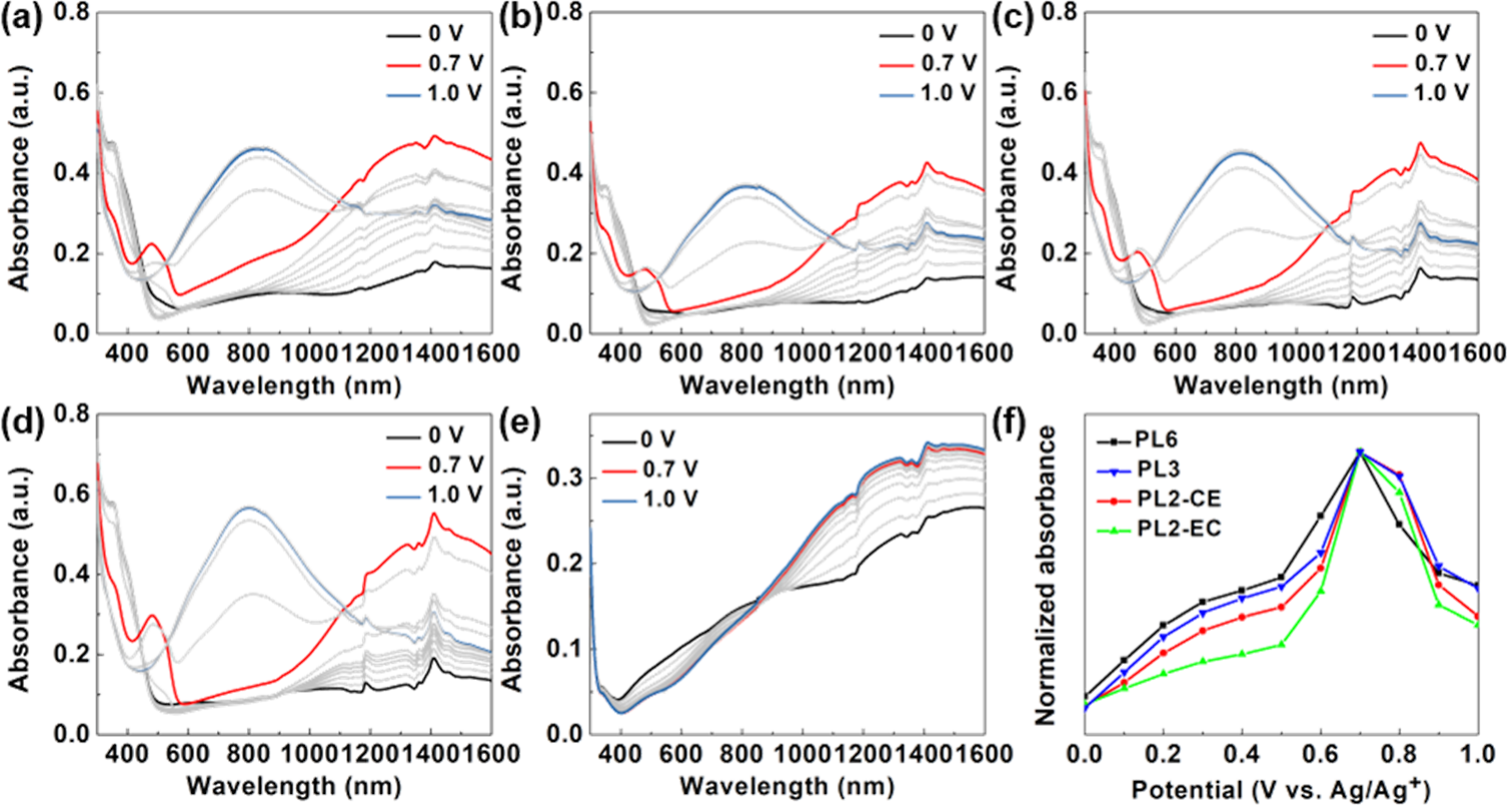
Spectroelectrochemistry of (a) PL6, (b) PL3, (c) PL2-CE, (d) PL2-EC, and (e) PEDOT with applied potentials from 0 to 1.1 V. (f) Normalized absorbance versus applied potential.

Figure 8 shows the UV–vis spectra of PL6, PL3, PL2-CE, and PL2-EC. All films exhibit significant differences compared to P8. PL6, PL3, PL2-CE, and PL2-EC were first discussed together as a group (PLs). From the UV–vis spectrum of PLs, a markedly large increase in NIR absorption can be observed under increasing potential compared to P10 and P8, displaying behavior similar to that of PEDOT (Figure 8e). Moreover, the absorption peak at 1400 nm in PLs matches the peak at 800 nm, corresponding to the formation of the TPB dication. Finally, the absorption at 300 nm shows a minimal decrease with increasing potential, a characteristic observed in CT, P10, and P8. All these features indicate a significant proportion of PEDOT in PLs compared to P10 and P8, suggesting that the growth of PEDOT is highly restricted by PCT segments in copolymerization. In contrast, PEDOT can be deposited on ITO with higher efficiency in the layer-by-layer method since no competitive reaction is involved. The optical properties of P8 and PLs were further compared under 0, 0.7, and 1.0 V. Previously, the absorbance in the NIR region was monitored at 0.6 V to compare CT, P10, and P8. However, for PLs, the absorbance noticeably increases from 0.6 to 0.7 V. Thus, PLs were compared with P8 at 0.7 V. The results were normalized at 316 nm, the characteristic peak of CT, to emphasize the PEDOT proportion in each sample (Figure S11). It is observed that the absorbance of PLs at each potential is higher than that of P8, increasing progressively from that of PL2-EC, PL2-CE, PL3, and PL6. These results indicate that better properties are achieved with an increase in the number of layers. Furthermore, at 0.7 V, the peak centered at 800 nm due to TPB dication formation is observed for P8, whereas PL6 shows a minimal peak, and the peaks for PL3, PL2-CE, and PL2-EC do not emerge until 0.8 V. This highlights another advantage of PLs: higher NIR absorbance can be achieved with a minimal increase in visible-region absorbance. This property is desirable for smart windows as it enables effective NIR absorption while preserving visible-light transmittance. An absorbance vs applied potential graph was plotted (Figure 8f). The absorbance of PLs was normalized to their respective maximum absorbance, showing that PL6 has equivalent absorbance increases at 0.6 and 0.7 V. The relative absorbance increased at 0.7 V becomes larger from PL6, PL3, PL2-CE, and finally to PL2-EC. This reflects that PL6 and PL3, which have lower oxidation potential, initiate the oxidation reaction sooner at a lower potential. Then, PL2-CE follows, and finally, PL2-EC, for which maximum increase in absorbance is obtained at 0.7 V. In conclusion, the results indicate that PL6 has the maximum increase in absorbance at 0.6 V and exhibits the largest absorbance at 1400 nm compared to other layer-by-layer structures with the same PCT proportion. Despite its complex fabrication steps, layer-by-layer polymerization offers a better method to construct the PCT–PEDOT electrochromic film without constraining the polymerization of both CT and BiEDOT.

The films prepared through copolymerization or the layer-by-layer approach in this study may appear to exhibit a similar behavior. However, this similarity arises due to the constraints imposed on the number of layers, cycles, and concentrations of CT and BiEDOT, which were adjusted to match the conditions of P8 for comparative purposes. Without these limitations, the layer-by-layer approach offers a wide range of tunable parameters, providing significant flexibility to control the spectroelectrochemical properties. An additional experiment was carried out to further illustrate the difference between the two methods. A film formed using the layer-by-layer method (two scans of CT for every scan of BiEDOT) was compared with P2. As shown in Figure S12, the film prepared by the layer-by-layer method exhibits a visible red color at 0.6 V, while P2 appears darker in blue. In this case, the film formed by the layer-by-layer approach is lighter since the growth of BiEDOT is not involved in every cycle.

**Figure 9:**
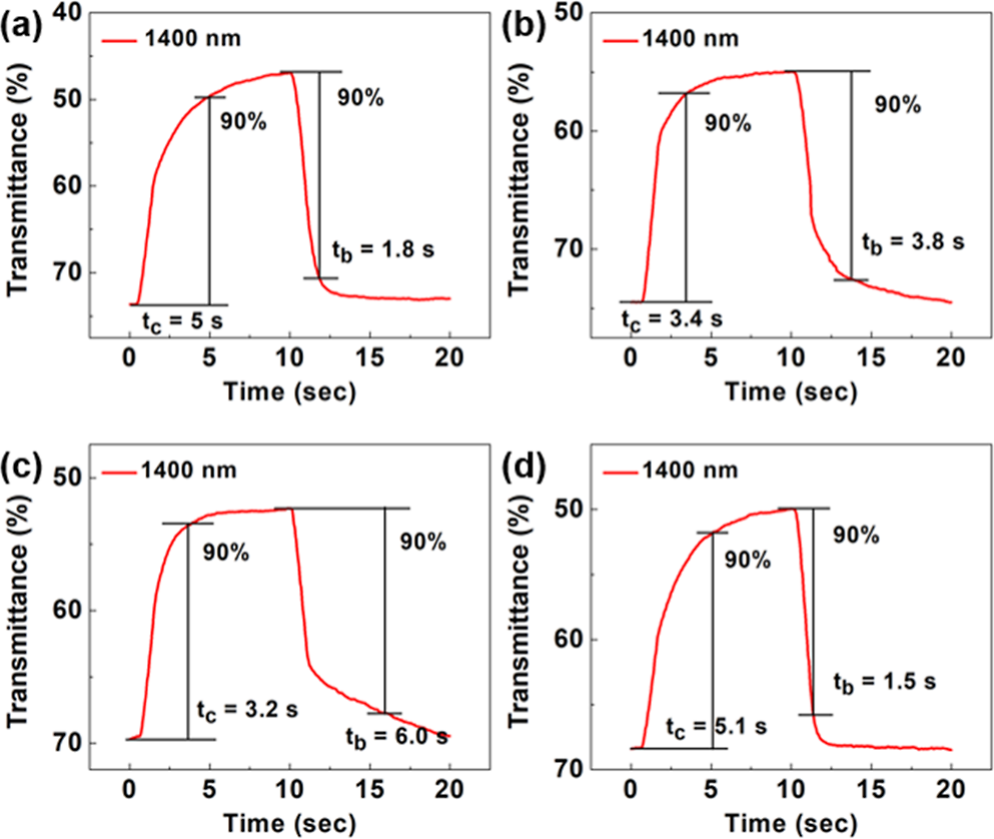
Coloration and bleaching time (*t*_c_ and *t*_b_) of (a) PL6, (b) PL3, (c) PL2-CE, and (d) PL2-EC at 1400 nm.

The electrochemical switching properties of PLs are shown in Figure 9 and Figure S13. The coloring times, *t*_c_, of PL6, PL3, PL2-CE, and PL2-EC under 0.6 V were 5, 3.4, 3.2, and 5.1 s, respectively. The bleaching times, *t*_b_, were 1.8, 3.8, 6, and 1.5 s, respectively. Under 1.0 V, the *t*_c_ values of PL6, PL3, PL2-CE, and PL2-EC were 4.7, 4.7, 5.4, and 3.8 s, respectively. The bleaching times, *t*_b_, were 1.9, 1.5, 1.7, and 1.5 s, respectively. In this study, the bleaching time (*t*_b_) is generally less than the coloration time (*t*_c_) across almost all systems. Notably, for PL2-CE monitored at 1400 nm, *t*_b_ was observed to be larger than *t*_c_. This phenomenon can be attributed to the mechanism wherein, during reduction, the outer PEDOT layer, which has a much lower reduction potential, impedes the reduction of the inner PCT layer. This hindrance results in a longer bleaching time than the coloration time for PL2-CE. Moreover, the cycling stability of PL6 was tested. After 200 switching cycles between 0 and 0.6 V, PL6 lost 0.5% of the optical contrast at 1400 nm. In the case of 800 nm, after 200 switching cycles between 0 and 1.0 V, PL6 lost 2% of the optical contrast. The stability of PL6 at 1400 nm was further evaluated over 500 switching cycles between 0 and 0.6 V. After 500 cycles, PL6 exhibited a 3% loss in optical contrast. The long-term electrochromic stability aligns closely with the performance reported in the literature,^60−66^ showing potential for practical applications.

## Conclusions

In this study, the electrochemical and electrochromic properties of polymers derived from CT and EDOT through copolymerization and layer-by-layer polymerization techniques were investigated. Due to the high oxidation potential of EDOT, an EDOT dimer (BiEDOT) was synthesized to lower the oxidation potential and enhance copolymerization with CT. Electrochemical analysis reveals that the introduction of BiEDOT significantly improves the redox properties and allows successful copolymerization, as evidenced by XPS results. The copolymer films P10 and P8 demonstrate enhanced electrochromic properties, with the P8 film showing the highest performance in terms of NIR absorption. On the other hand, layer-by-layer polymerization yields films with distinct electrochemical behaviors compared to copolymerized films. Additionally, the properties can be altered by changing the number of cycles and the sequence of deposition. UV–vis spectroscopy further confirms that layer-by-layer films exhibit a higher NIR absorbance and better optical properties than copolymerized films. Overall, the study demonstrates the effectiveness of the innovative layer-by-layer polymerization method to construct PCT–PEDOT electrochromic films with better electrochromic performance and higher NIR absorption compared to copolymerization.

## Supporting Information Available

NMR spectra of CT and BiEDOT; XPS profiles, SEM images, and contact angles of the copolymers; electropolymerization data, cyclic voltammograms, and electrochromic switching for CT-BiEDOT copolymer films; and UV–vis of PLs (PDF)

## Abbreviations

CT: corannulene-(triphenylamine)_5_
EDOT: 3,4-ethylenedioxythiophene
BiEDOT: 2,2′-bis(3,4-ethylenedioxy) thiophene
NMR: nuclear magnetic resonance
XPS: X-ray photoelectron spectroscopy
PC: propylene carbonate
DCM: dichloromethane
NIR: near-infrared
UV–vis: ultraviolet–visible spectroscopy
CV: cyclic voltammetry
SEM: scanning electron microscopy
EIS: electrochemical impedance spectroscopy
