# Supplementary material for: Enhanced NIR Electrochromic Properties of Corannulene-(triphenylamine)5 and EDOT-Derived Polymers via Electrochemical Layer-by-Layer Polymerization Compared to Copolymerization
Source: ACS Appl Mater Interfaces. 2025 Feb 5;17(7):10920–30. doi: 10.1021/acsami.4c21400 (PMC11843537; doi:10.1021/acsami.4c21400)
Supplement: Supplementary file 1 — am4c21400_si_001.pdf [file am4c21400_si_001.pdf]

Supporting Information

Enhanced NIR Electrochromic Properties of Corannulene-(triphenylamine)$_5$ and EDOT-Derived Polymers via Electrochemical Layer-by-Layer Polymerization Compared to Copolymerization

Chiao-Ling Yu and Shyh-Chyang Luo*

Department of Materials Science and Engineering, National Taiwan University, No. 1, Sec. 4, Roosevelt Road., Taipei 10617, Taiwan

*Email: shyhchyang@ntu.edu.tw

Table of Contents

Figure S1: ^{1}H-NMR of Corannulene-(triphenylamine)$_5$ in CD_2Cl_2.
Figure S2: ^{1}H-NMR of 2,2'-bis-(3,4-ethylenedioxy)thiophene in $(CD_3)_2S{=}O$).
Figure S3: CV diagram of corannulene.
Figure S4: XPS results of (a) N1s spectra, (b) S2p spectra for CT-EDOT copolymerization.
Figure S5: Contact angle of ITO, PCT, P10, P8, and PEDOT.
Figure S6: XPS results of (a) N1s spectra, (b) S2p spectra for CT-BiEDOT copolymerization.
Figure S7: CV diagrams of (a) PCT, (c) P10, and (e) P8 films with varying scan rate and the plot of current versus square root of scan rate for oxidation and reduction peaks of (b) PCT, (d) P10, and (f) P8.
Figure S8: Electrochromic switching of (a) PCT, (b) P10, and (c) P8 at 1400 nm. And (d) PCT, (e) P10, and (f) P8 at 800 nm for 200 continuous cycles, and additionally, (g) P8 at 1400 nm for 500 continuous cycles.
Figure S9: Preparation of (a) PL6, (b) PL3, (c) PL2-CE, and (d) PL2-EC by conducting CV in CT and BiEDOT solutions alternatively.
Figure S10: CV diagrams of (a) PL6, (c) PL3, (e) PL2-CE, and (g) PL2-EC films with varying scan rate and the plot of current versus square root of scan rate for oxidation and reduction peaks of (b) PL6, (d) PL3, (f) PL2-CE, and (h) PL2-EC.
Figure S11: Normalized absorbance of PLs at (a) 0 V, (b) 0.7 V, and (c) 1.0 V
Figure S12: Color of (a) P2 and (b) PL (two scans of CT for every scan of BiEDOT) at 0, 0.6, and 1.0 V. The figure on the right demostrates the structure of PL.
Figure S13: The coloration and bleaching time (tc and tb) of (a) PL6, (b) PL3, (c) PL2-CE, and (d) PL2-EC at 800 nm. Electrochromic switching of PL6 at (e) 1400 nm and (f) 800 nm for 200 continuous cycles, and additionally, (g) PL6 at 1400 nm for 500 continuous cycles.

.

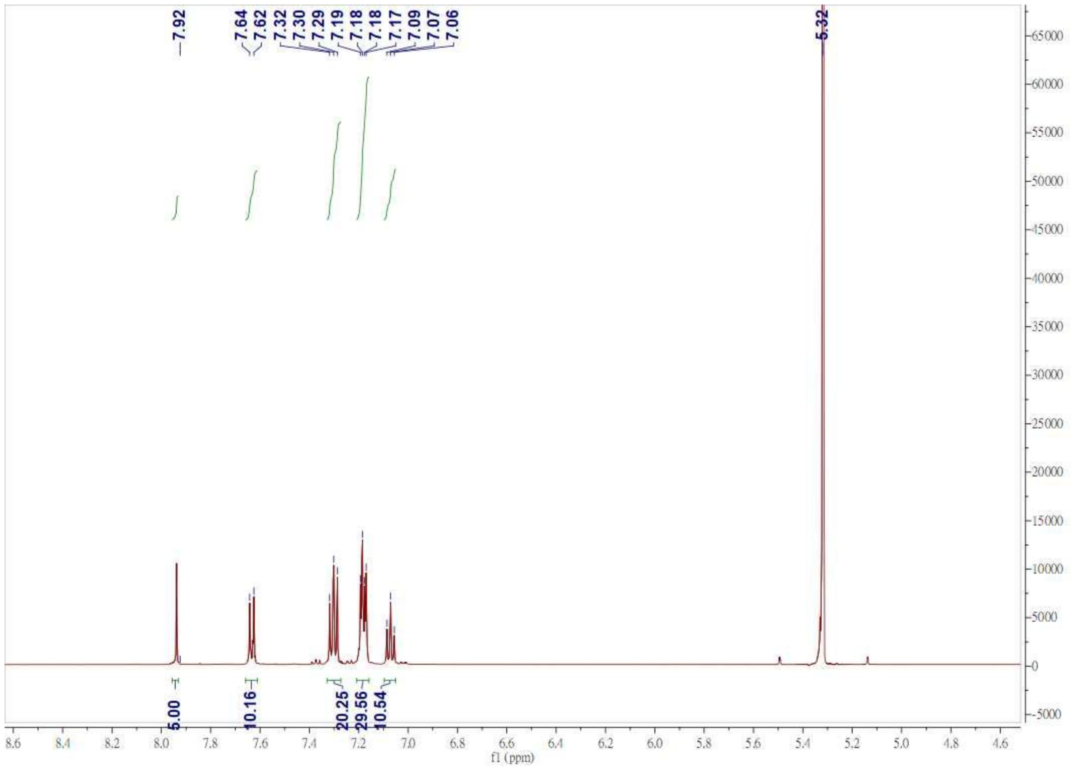

Figure S1. ^{1}H-NMR of Corannulene-(triphenylamine)$_5$ in CD_2Cl_2.

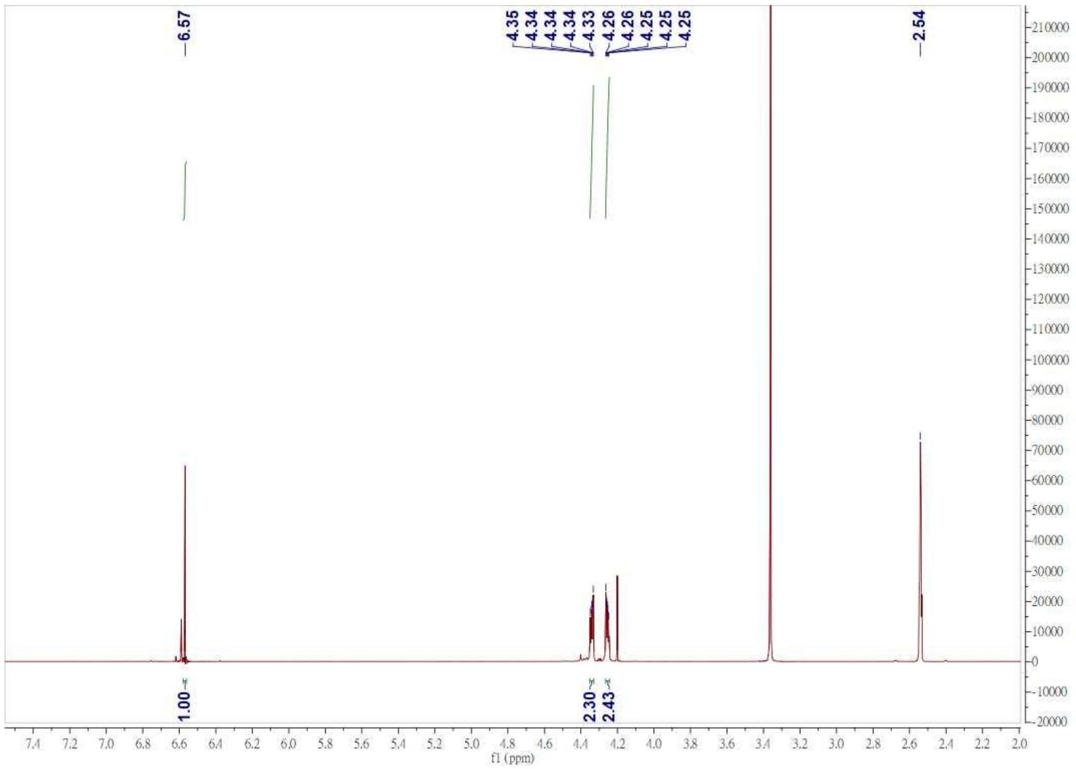

Figure S2. ^{1}H-NMR of 2,2'-bis-(3,4-ethylenedioxy) thiophene in $(CD_3)_2S$=O).

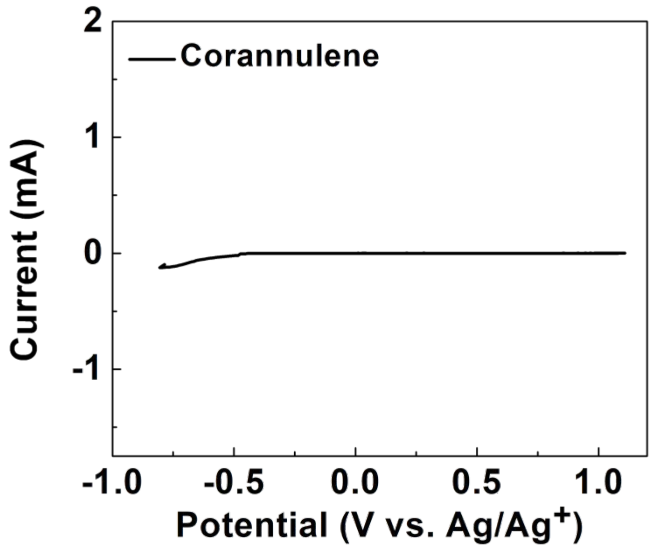

Figure S3. CV diagram of corannulene.

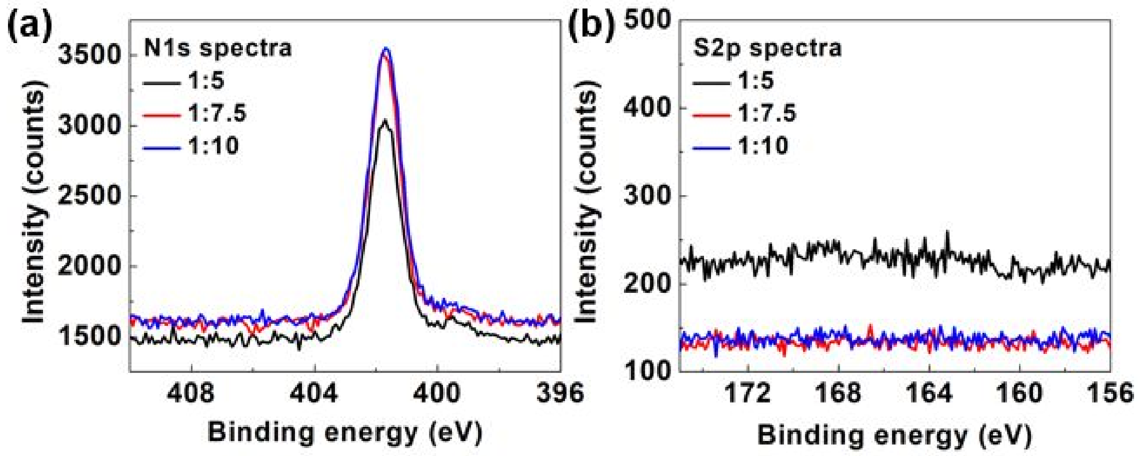

Figure S4. XPS results of (a) N1s spectra, (b) S2p spectra for CT-EDOT copolymerization.

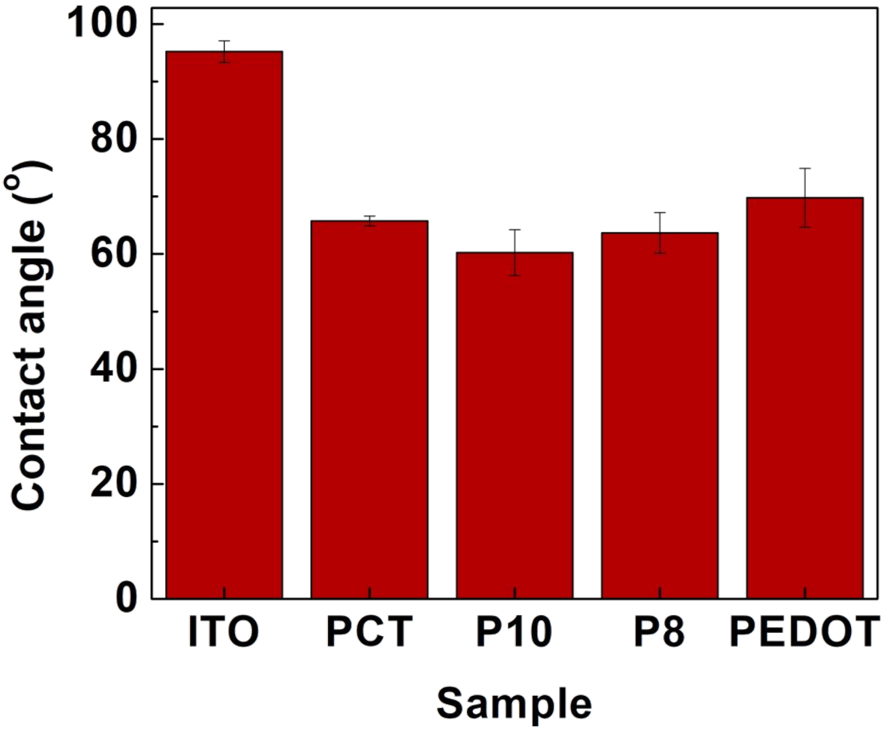

Figure S5. Contact angle of ITO, PCT, P10, P8, and PEDOT.

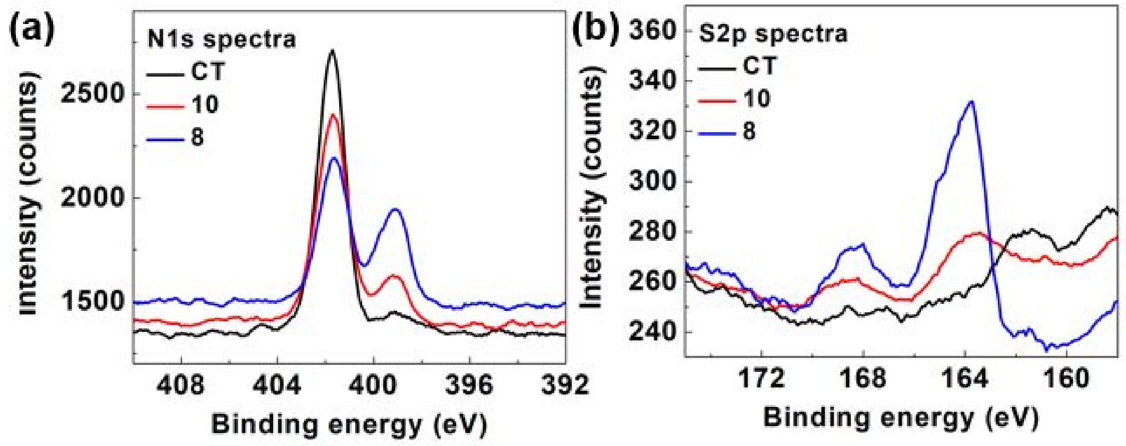

Figure S6. XPS results of (a) N1s spectra, (b) S2p spectra for CT-BiEDOT copolymerization.

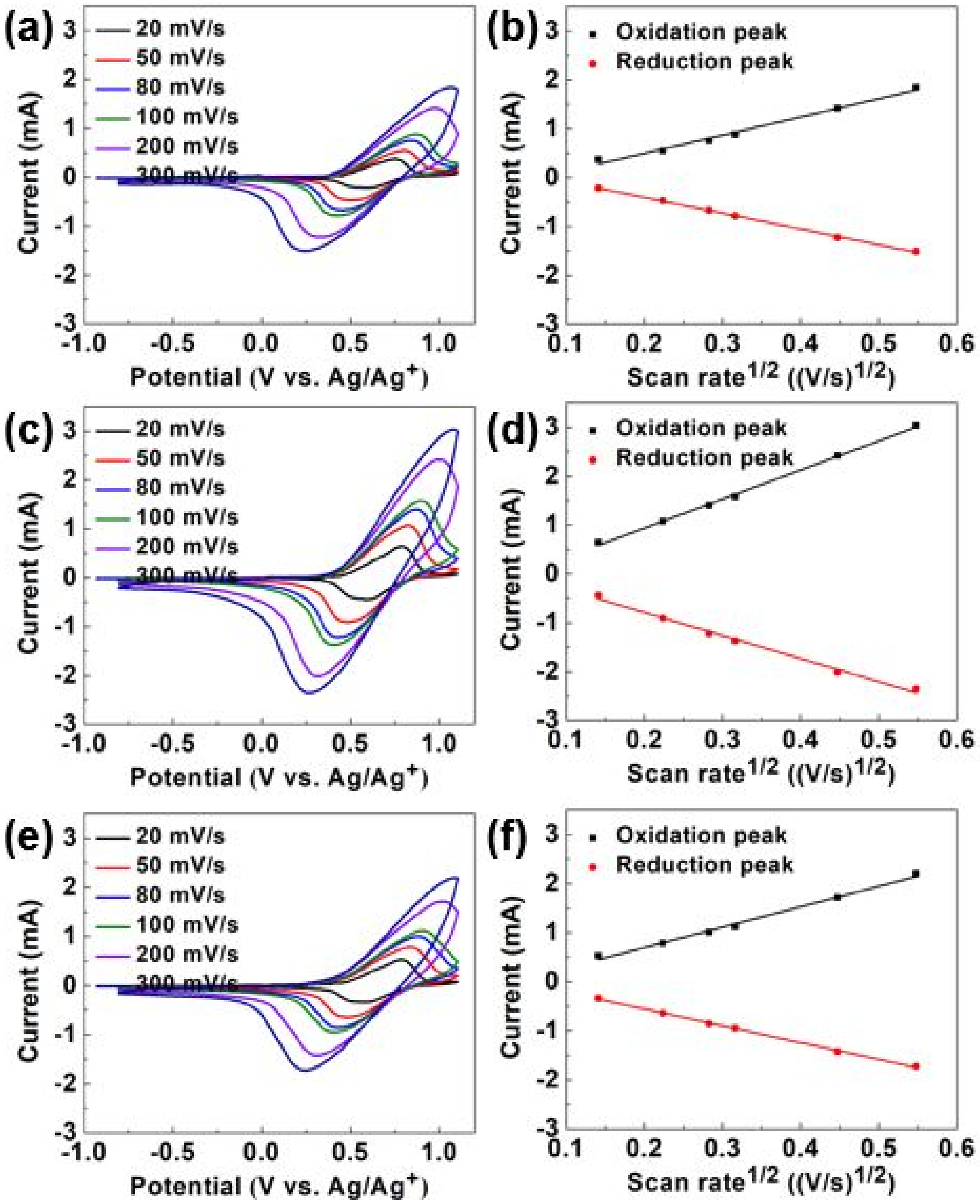

Figure S7. CV diagrams of (a) PCT, (c) P10, and (e) P8 films with varying scan rate and the plot of current versus square root of scan rate for oxidation and reduction peaks of (b) PCT, (d) P10, and (f) P8.

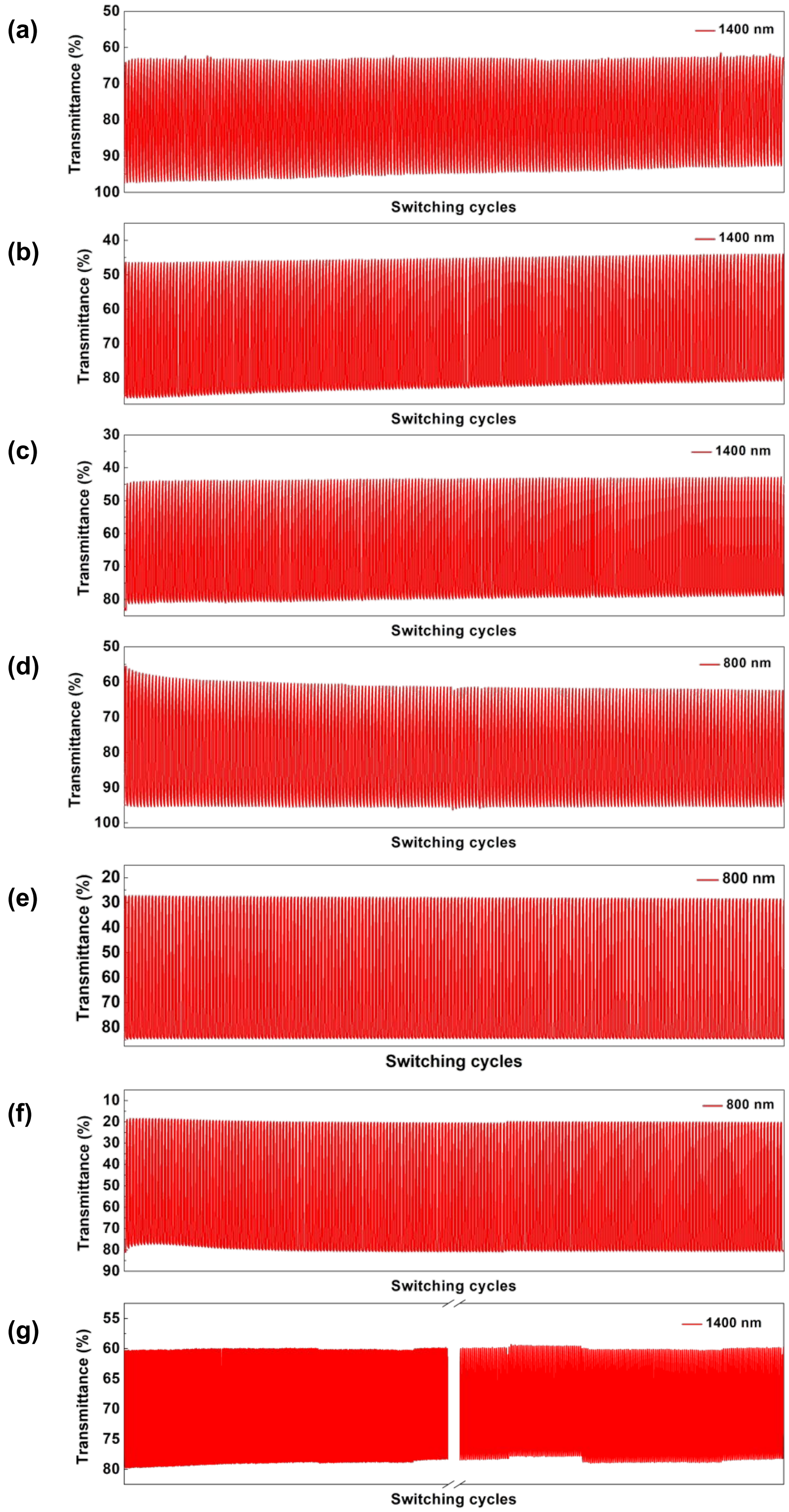

Figure S8. Electrochromic switching of (a) PCT, (b) P10, and (c) P8 at 1400 nm. And (d) PCT, (e) P10, and (f) P8 at 800 nm for 200 continuous cycles, and additionally, (g) P8 at 1400 nm for 500 continuous cycles.

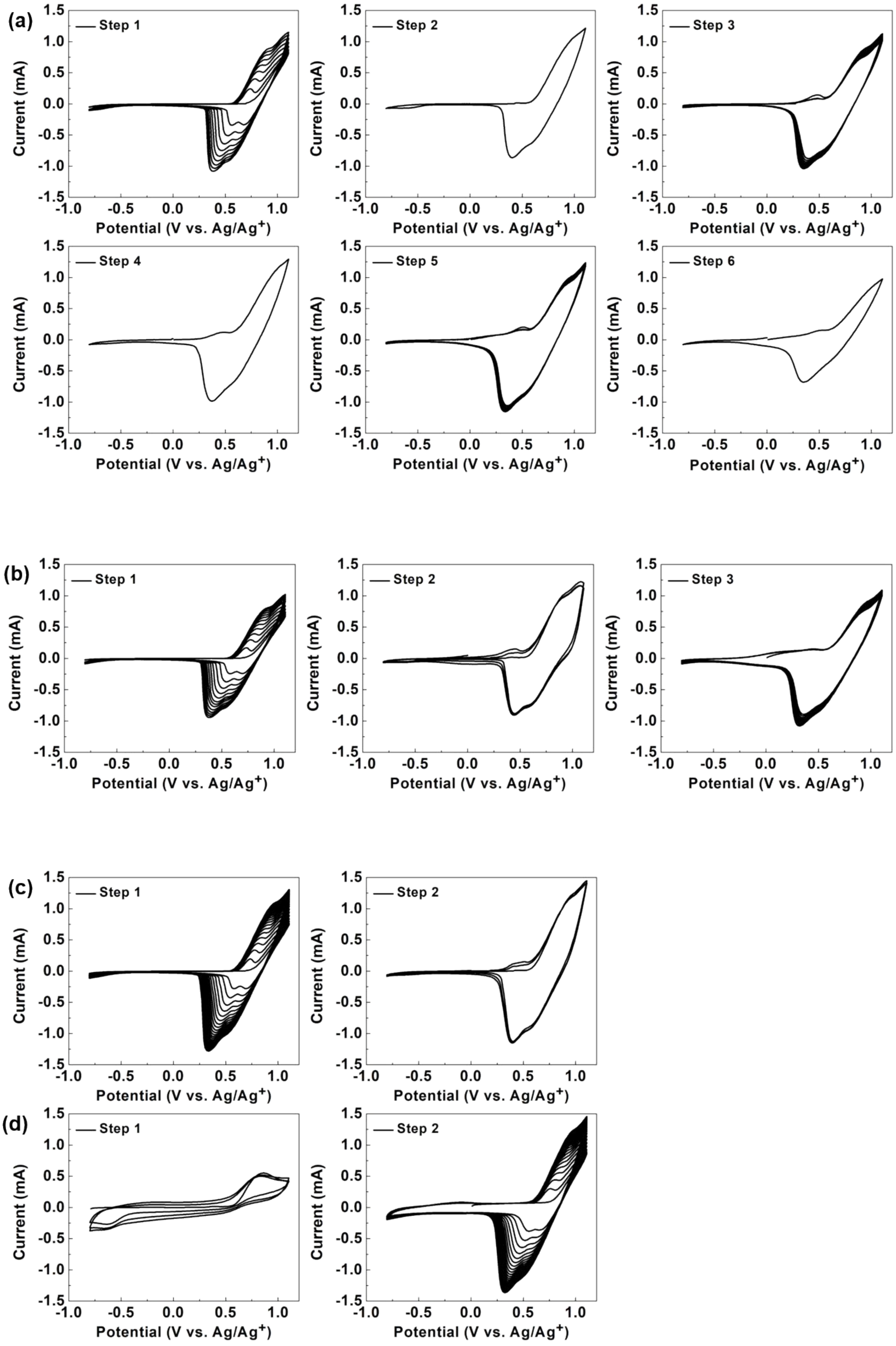

Figure S9. Preparation of (a) PL6, (b) PL3, (c) PL2-CE, and (d) PL2-EC by conducting CV in CT and BiEDOT solutions alternatively.

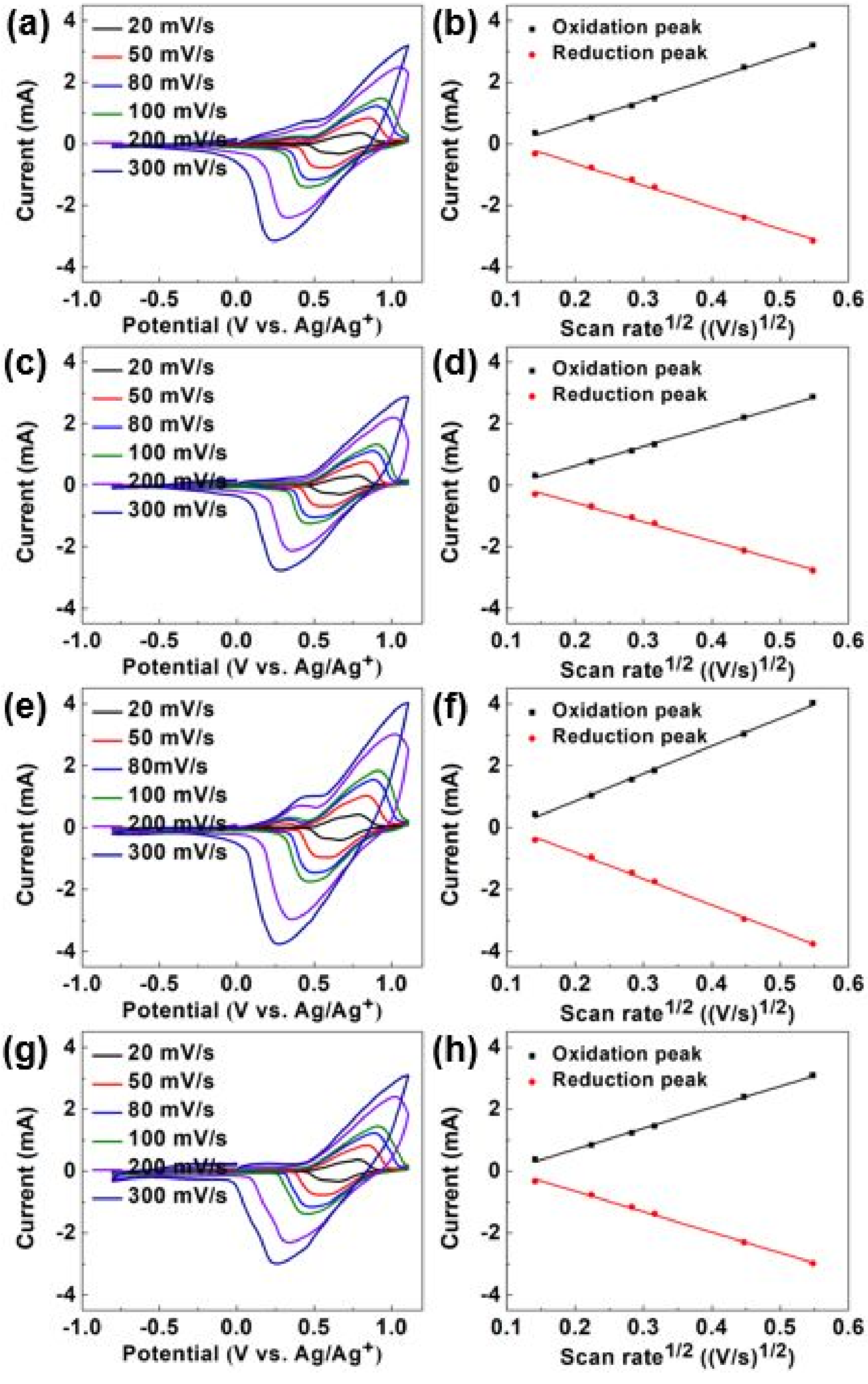

Figure S10. CV diagrams of (a) PL6, (c) PL3, (e) PL2-CE, and (g) PL2-EC films with varying scan rate and the plot of current versus square root of scan rate for oxidation and reduction peaks of (b) PL6, (d) PL3, (f) PL2-CE, and (h) PL2-EC.

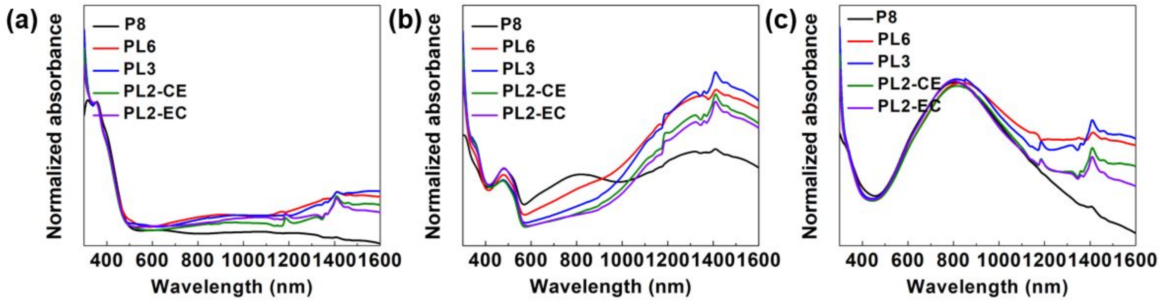

Figure S11. Normalized absorbance of PLs at (a) 0 V, (b) 0.7 V, and (c) 1.0 V

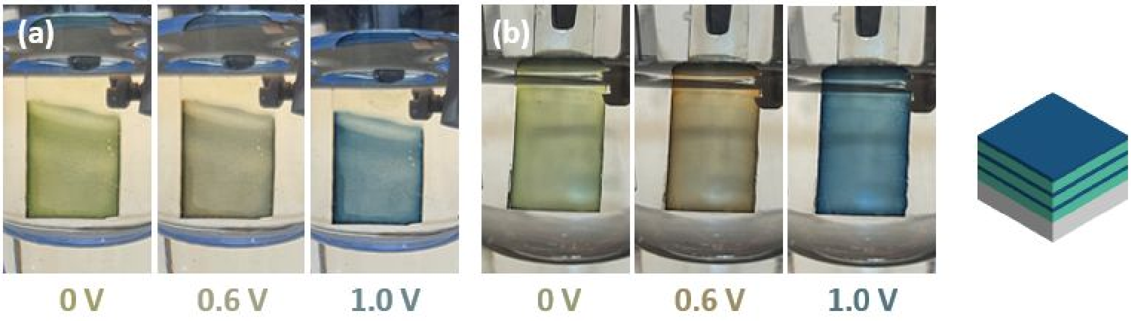

Figure S12. Color of (a) P2 and (b) PL (two scans of CT for every scan of BiEDOT) at 0, 0.6, and 1.0 V. The figure on the right demostrates the structure of PL.

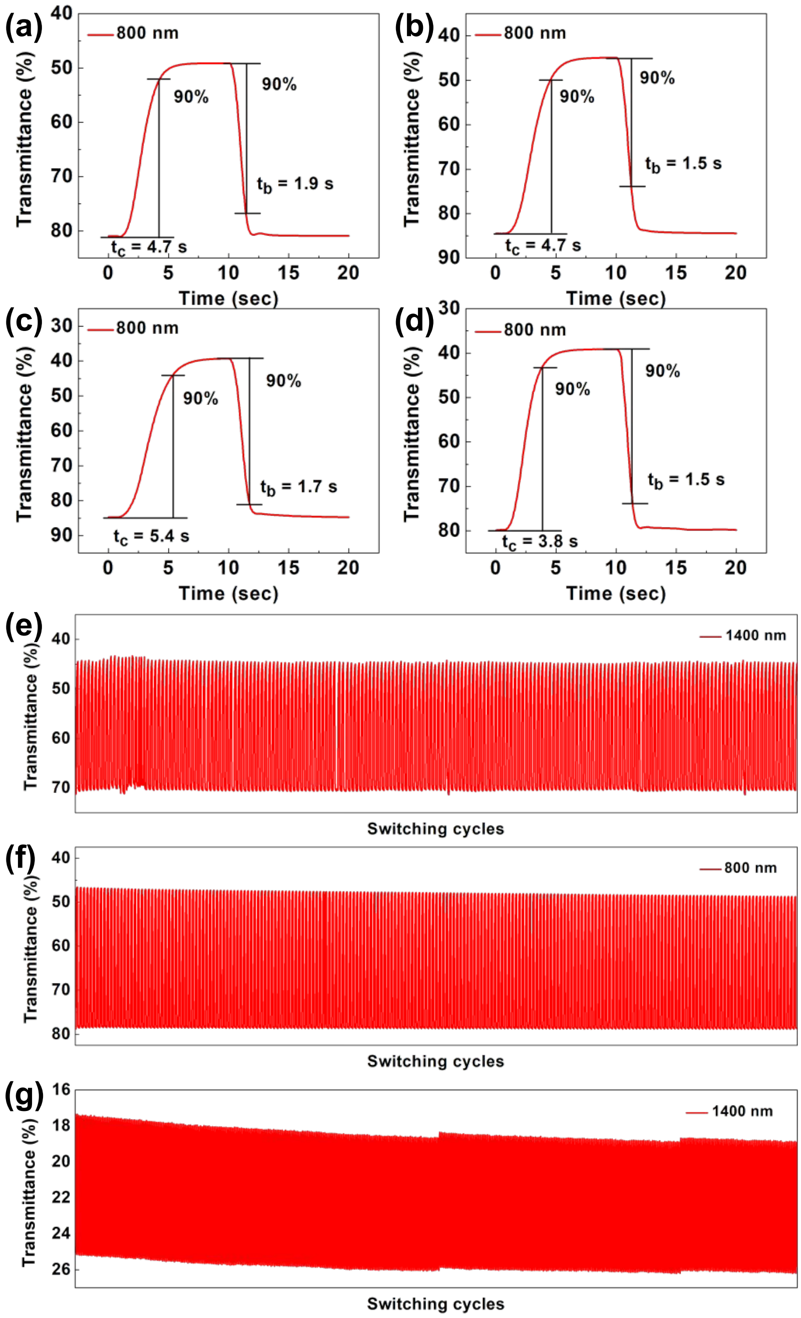

Figure S13. The coloration and bleaching time (t_c and t_b) of (a) PL6, (b) PL3, (c) PL2-CE, and (d) PL2-EC at 800 nm. Electrochromic switching of PL6 at (e) 1400 nm and (f) 800 nm for 200 continuous cycles, and additionally, (g) PL6 at 1400 nm for 500 continuous cycles.
